# A Novel DNA Methyltransferase *Dnmt3a3* Splice Variant Represses Preadipocyte Proliferation and Differentiation

**DOI:** 10.3389/fgene.2020.00115

**Published:** 2020-02-25

**Authors:** Bahareldin Ali Abdalla, Zhenhui Li, Qinghua Nie

**Affiliations:** ^1^ Department of Animal Genetics, Breeding and Reproduction, College of Animal Science, South China Agricultural University, Guangzhou, China; ^2^ National-Local Joint Engineering Research Center for Livestock Breeding, Guangdong Provincial Key Lab of Agro-Animal Genomics and Molecular Breeding and the Key Lab of Chicken Genetics, Breeding and Reproduction, Ministry of Agriculture, Guangzhou, China

**Keywords:** *Dnmt3a3*, alternative splicing, gene expression, cell cycle, preadipocyte differentiation

## Abstract

Proliferation and differentiation of preadipocyte are essential for the formation of fat tissues. However, the genes that regulate the early stage of preadipocyte differentiation in chicken have remained elusive. Here we identify a novel spliced variant of the DNA methyltransferase *Dnmt3a* gene, named *Dnmt3a3*, that controls early preadipocyte differentiation. *Dnmt3a3* expression is increased at the onset of preadipocyte differentiation and remains elevated during differentiation. Overexpression of *Dnmt3a3* in preadipocytes markedly inhibits proliferation and cell-cycle progression, and this is accompanied by inhibition of the mRNA and protein level of cell-cycle control genes, such as p21 and p27. In addition, forced expression of *Dnmt3a3* in differentiating preadipocytes represses early preadipocyte differentiation, and this was found to be accompanied by inhibition of the mRNA expression levels of early preadipocyte differentiation markers, such as *GATA2*, *GATA3*, *C/EBPα*, *C/EBPβ*, *AP2*, and *PPARγ*, or the protein levels of GATA3, C/EBP*β*, and PPAR*γ*. Taken together, these data demonstrate the participation of *Dnmt3a3* in the proliferation and differentiation process of chicken primary preadipocyte cells.

## Introduction

Preadipocyte proliferation and differentiation play an essential role in forming adipose tissue. The adipose tissue has a key role in energy homeostasis and also serves as an endocrine organ (Spiegelman and Flier, 1996). The fast growth of modern broilers has led to a series of developmental and metabolic syndromes, such as excessive abdominal fat accumulation (Abdalla et al., 2018a) and obesity (Siegel, 2014; Abdalla et al., 2018a). Therefore, studying the molecular genetics and cellular roles underlying adipose tissue development would improve our understanding of developmental and metabolic syndromes. Recently it has been reported that a number of transcription factors [*e.g.*, peroxisome proliferator-activated receptor-*γ* (*PPARγ*), transcription coactivators CCAAT/enhancer binding protein *α* (*C/EBPα*) and CCAAT/enhancer binding protein *β* (*C/EBPβ*)] and genes [*e.g.*, adipocyte fatty-acid binding protein (*AP2*; also known as *FABP4*) and glucose transporter protein (*GLUT4*)] are involved in early preadipocyte differentiation (reviewed by Ghaben and Scherer, 2019). Regulation of preadipocyte proliferation and differentiation is associated with the expression of specific cell cycle regulator genes (Morrison and Farmer, 1999). The cell cycle is the sequence of events in which one cellular component is doubled and then accurately separated into two daughter cells. The central machines that drive cell cycle progression are the cyclin-dependent kinases (*CDKs*) belonging to the *CIP/Kip* family (Morrison and Farmer, 1999), such as *p21* (also known as *Cdkn1a* and *Cip1*) and *p27* (also known as *Cdkn1b* and *kip1*). These are able to phosphorylate key substrates to promote DNA synthesis and mitotic progression.

DNA methyltransferase 3a (*Dnmt3a*) gene was first identified in human and mouse and is highly expressed in undifferentiated embryonic stem cells, but at a lower level in differentiated cells and adult somatic tissues (Okano et al., 1998). Mice lacking *Dnmt3a* died at around 4 weeks of age (Okano et al., 1999). *Dnmt3a* is essential for establishing global-*de-novo* methylation (rather than maintenance methylation) mainly at cytosine-phosphate diester-guanine dinucleotides in embryonic stem cells and during embryogenesis (Okano et al., 1999). A previous study indicated that *Dnmt3a* mRNA expression is increased in the adipose tissue of obese mice (Kamei et al., 2010).

Splicing of mRNA precursors to mature mRNAs is a highly dynamic and complex phenomenon affecting development in animals and plants, and it has received increasing attention over recent years (Brett et al., 2002; Blencowe, 2006; Wang et al., 2019). Two isoforms of *Dnmt3a, Dmnt3a* (long) and *Dnmt3a2* (short), have been identified in human and mouse (Chen et al., 2002; Weisenberger et al., 2002). The structural differences between these two isoforms occur at the N-terminal end of the proteins (Chen et al., 2002). *Dnmt3a2* is predominant in embryonic stem cells and is mainly localized to euchromatin, whereas *Dnmt3a* is primarily concentrated in the heterochromatin (Chen et al., 2002). Previously, we identified two transcripts of *Dnmt3a*, namely *Dnmt3a* and *Dnmt3a1*, in chicken (Abdalla et al., 2018b). The chicken *Dnmt3a1* transcript was highly expressed in adipose tissue/cell (Abdalla et al., 2018b) and its overexpression inhibits adipogenesis. However, the biological roles of many spliced variants of a gene are not fully understood.

In this study, we report the identification of a novel spliced variant of the *Dnmt3a* gene, named *Dnmt3a3*, that controls early preadipocyte differentiation. *Dnmt3a3* mRNA level is increased at the onset of preadipocyte differentiation and remained elevated during the time course of differentiation. Moreover, overexpression of *Dnmt3a3* inhibits preadipocyte proliferation and differentiation. Overall, our data identified and highlighted the potential role of *Dnmt3a3* transcript in modulating adipogenesis.

## Materials and Methods

### Animals and Ethics Statement

A young-female group (25-day-old; *n* = 4) and an adult-female group (350-day-old; *n* = 4) of yellow-feathered chickens and a young-female group (25-day-old; *n* = 4) of Huaixiang chickens (a local Chinese breed) were obtained from a commercial chicken farm (Kwangfeng, Guangzhou, China). Adipose tissues (abdominal fat and subcutaneous fat) were carefully collected, immediately snap frozen in liquid nitrogen, and stored at −80°C until use. All experimental procedures involving chickens in this study were approved by the Institutional Animal Care and Use Committee of the South China Agriculture University (Approval Number SYXK2014-0136) and efforts were made to minimize pain or discomfort of the birds.

### Primary Preadipocyte Isolation and 
*In Vitro* Differentiation

Chicken primary preadipocytes were isolated from the abdominal fat tissue of 25-day-old yellow-feathered chicks (Kwangfeng) under sterile conditions as previously reported (Abdalla et al., 2018b). Briefly, the chicks (*n =* 3) were humanely slaughtered and the abdominal fat tissues (about 3 g) were rapidly excised, placed in a 6-cm petri dish containing 4 ml DMEM/F12 medium (Invitrogen, Carlsbad, United States), and minced into sections of approximately 1 mm^2^ using scissors. To release single cells, the suspension was digested with collagenase (Invitrogen) for 30 min at 37°C. The single cells were then collected by centrifugation at 1,500 g for 5 min. The cell pellets were resuspended in normal growth (proliferation) medium (DMEM/F12 supplemented with 15% (v/v) fetal bovine serum (FBS) (HyClone, Logan, United States) and 1% penicillin/streptomycin (Invitrogen)). The cells were cultured in 5% CO_2_ atmosphere at 37°C.

To induce adipogenic differentiation, the cells were seeded at a density of 4 × 10^4^ cells per cm^2^ in 12-well plates and incubated for 24 h before switching to adipogenic (differentiation) medium (DMEM/F12 supplemented with 10% FBS (vol/vol), 1% penicillin/streptomycin (Invitrogen), and 0.04 mM oleate (Sigma-Aldrich, St. Louis, United States)). Adipogenesis was assessed using quantitative RT-PCR (qRT-PCR) analysis of differentiation markers, such as *PPARγ*, *C/EBPα*, and *C/EBPβ* and Western blot analysis of PPAR*γ* and C/EBP*β*. The lipid accumulation in differentiating preadipocytes was visualized using Oil-Red-O staining kit (Solarbio, Beijing, China) according to the manufacturer’s instructions. Images were captured using a Leica DM2000 LED microscope (Leica, Wetzlar, Germany).

### RNA Extraction, Complementary DNA (cDNA) Synthesis, and qRT-PCR Analysis

Total RNA was extracted from tissues or cells using RNAiso Plus reagent (TaKaRa, Kusatsu, Japan) according to manufacturer’s instructions. The integrity of isolated RNA was analyzed by agarose gel (1.5%) electrophoresis. The RNA concentration was determined by measuring the optical density (OD) in a NanoDrop One spectrophotometer (Thermo Scientific, Wilmington, United States) at 260/280 nm ratio.

Equal amounts (1.2 µg) of RNA were reverse transcribed into cDNA using a PrimeScript RT Reagent Kit with gDNA Eraser (TaKaRa) according to the manufacturer’s protocol.

The qRT-PCR primers were designed using OLIGO Primer Analysis Software v. 7 (Molecular Biology Insights, Colorado Springs, United States) and synthesized by TSINGKE Biological Technology Co., Ltd. (Beijing, China). qRT-PCR analysis was performed as follows: 1 µl of cDNA (10 ng equivalent) was added to a reaction composed of 5 µl SYBR^®^ Green master mix (Bio-Rad, Irvine, United States), 0.5 µl of 5 µM primers and nuclease-free water to a final volume of 10 µl. DNA amplification (predenaturation for 3 min at 95°C and 40 cycles of 95°C for 5 s, 58°C for 38 s, and 65°C for 5 s followed by melting curve analysis) was monitored using a CFX96 Real-Time Detection instrument (Bio-Rad). All reactions were run in triplicate. Glyceraldehyde-3-phosphate dehydrogenase (*GAPDH*) gene was used as an internal control (Gonzales et al., 2015). The expression data were analyzed using the relative quantification method 2-^ΔΔCT^. The primer pairs used for qRT-PCR analyses are listed in **Table 1**.

**Table 1:** Primer sequences for qRT-PCR.

Gene	Sequence (5′ to 3′）	Size (bp)	Accession number
*Dnmt3a*	Forward: AGGGCACTGGGCGCCTCTTCTT	174	LC381635.1
	Reverse: TGGCATCAATCATGACGGGGTT		
*Dnmt3a1*	Forward: GATGAGGGCAAGGAGGAGAAG	314	LC379990.1
	Reverse: TCGGTGGGCTGCTGTGAG		
*Dnmt3a3*	Forward: GGGCCGGGCACTGGGCGCCTCT	178	LC469746.1
	Reverse: TGGCATCAATCATGACGGGGTT		
*p21*	Forward: ACGCGGGCAGACCACCATCAAA	190	NM_204396.1
	Reverse: AGGGAACTACAGACTCGGCATT		
*p27*	Forward: AGCAAACACCCAAGAAATCGAG	148	NM_204256.2
	Reverse: ATGGAGACCGACGATATGTATT		
*Cyclin D1*	Forward: CGGTGTCCTACTTCAAGTGCGT	276	NM_205381.1
	Reverse: TGATGGAGTTGTCGGTGTAAAT		
*PPARγ*	Forward: AGATTACAATGGTTGACACAGA	338	NM_001001460.1
	Reverse: ACGACATTCAATAGCCATAAGT		
*C/EBPα*	Forward: TGGAGCAAGCCAACTTCTACGA	114	NM_001031459.1
	Reverse: AGGGAGCCTCTCTGTAGCCGTA		
*C/EBPβ*	Forward: CCGTCTTCTCCTCCCCGCATCC	233	NM_205253.2
	Reverse: AGGGGCTGAAGTCAATGGCTCT		
*AP2* *(FABP4)*	Forward: GACTATATGAAAGAGCTGGGTG	183	NM_204290.1
	Reverse: TCTGTCATCTGCTGTGGTCTCA		
*GATA2*	Forward: AATGCCTGCGGACTCTACTATA	270	NM_001003797.1
	Reverse: GTGGATGGGGGTAGGTGTTGGT		
*GATA3*	Forward: GATCCTGTCTGTAATGCCTGTG	222	NM_001008444.1
	Reverse: GGGTGAAATGTGGCTGATAGAG		
*Pref-1*	Reverse: GGGTGAAATGTGGCTGATAGAG	214	NM_001142254.1
	Forward: CAACTTTGGCATTGTGGAGG		
*GAPDH*	Forward: CAACTTTGGCATTGTGGAGG	130	NM_204305.1
	Reverse: CGCTGGGATGATGTTCTGG		

### Detection of Novel Splice Variants of *Dnmt3a* Gene

The novel transcript detection of *Dnmt3a* was performed as previously described (Abdalla et al., 2018b). Briefly, PCR amplification and Sanger sequencing methods were used to detect novel splice variants of *Dnmt3a*. The gene sequence encoding *Dnmt3a* was retrieved from the NCBI database. We designed a primer pair for *Dnmt3a* (GenBank accession no. NM_001024832.1) spliced variant detection using OLIGO Primer Analysis Software v. 7 (Molecular Biology Insights) as shown in **Table 2**. Total RNA was prepared from the abdominal fat tissue of 25-day-old Huaixiang chicks (*n =* 4) by RNAiso Plus reagent (TaKaRa), while cDNA was synthesized from 1.2 µg of total RNA using a PrimeScript RT Reagent Kit with gDNA Eraser (TaKaRa). The full-length coding sequence of *Dnmt3a* was amplified by PCR from the pooled abdominal fat tissue cDNA (four samples) from Huaixiang chicks using the primer pair described above. The PCR amplification of the pooled cDNA was performed in a volume of 50 µl using KOD FX kit (Toyobo, Osaka, Japan) following the manufacturer’s instructions. DNA amplification (predenaturation for 2 min at 94°C, followed by 38 cycles of denaturation at 98°C for 10 s, annealing at 60°C for 30 s, and extension at 68°C for 2.8 min, and finally extension at 68°C for 7 min) was monitored using a Takara PCR Thermal Cycler instrument (Takara). PCR products were gel purified using HiPure Gel Pure DNA Micro Kit (Magen, Guangzhou, China) and sequenced by TSINGKE Biological Technology Co., Ltd. (Beijing, China). The sequences were analyzed using the SeqMan procedure of DNAStar software and BLAST (Basic Local Alignment Search Tool) database to identify a novel splice variant of *Dnmt3a*.

**Table 2:** Primer sequences for spliced variant detection and plasmid construction.

Primer names	Sequence (5′ to 3′)
*Dnmt3a* spliced variant detection	Forward: TGCGCCATGGTGGAAAGCAGTGACACACCC
	Reverse: GGCACAGTCCCCGTCCGGCTCTCCTA
pSDS-*Dnmt3a3*	Forward: **GGGGggtctctagtg**TGCGCCATGGTGGAAAGCAGTGA CACACCC
	Reverse: **GCCGggtctcgtggg**GGCACAGTCCCCGTCCGGCTCTCCTA

Sequences in bold represent the BsaI restriction sites.

### Construction of *Dnmt3a3* Overexpression Plasmid and Transient Transfection

The *Dnmt3a* cDNA sequence (GenBank accession no. NM_001024832.1) from the chicken was amplified by PCR from the pooled cDNA (as described above) of Huaixiang chicks using the *Dnmt3a*-specific primer pair, named pSDS-*Dnmt3a3* primer (**Table 2**), and then the products of amplification were sequenced. After sequence confirmation, the specific band of *Dnmt3a3* in the gel was eluted using HiPure Gel Pure DNA Micro Kit (Magen) and cloned into pSDS-25202 expression vector (SiDanSai, Shanghai, China) using BsaI restriction sites. The constructed plasmid was confirmed through sequencing and named pSDS-*Dnmt3a3* (overexpression). The pSDS-25202 empty vector was used as a negative control and termed pSDS. Transient transfections of preadipocytes were carried out with Lipofectamine 3000 transfection reagent (Invitrogen) according to the manufacturer’s instructions. To confirm the *Dnmt3a3* overexpression efficiency, 210,000 preadipocytes were seeded in 12-well plates in normal growth medium (as described above) in 5% CO_2_ atmosphere at 37°C. The following day, the cells were transfected with pSDS-*Dnmt3a3* or pSDS using Lipofectamine 3000 (Invitrogen). 48 h after the initial transfection, the cells were harvested and immediately subjected to total RNA extraction (as noted above). The extracted total RNA was reverse transcribed to cDNA, and then qRT-PCR was performed to confirm the overexpression efficiency.

### Flow Cytometric Analysis of Cell Cycle

Flow cytometry experiments were performed to analyze the cell cycle distribution in proliferating and differentiating preadipocytes after *Dnmt3a3* overexpression or negative control using the Cell Cycle and Apoptosis Analysis kit (Beyotime, Jiangsu, China) according to the manufacturer’s protocols. The cells were cultured in 12-well plates (19,000 cells per well) in normal growth medium (as described above) for 48 h after transfection or in growth medium for 6 h after transfection and then transferred to adipogenic medium (as described above) for 48 h. At 80% confluency, the cells were transfected with pSDS-*Dnmt3a3* or pSDS using Lipofectamine 3000 (Invitrogen). After transfection for 48 or 54 h, the cells were detached with trypsin, washed once with phosphate buffered saline (PBS), and fixed in 66% ethanol at 4°C. Following 24 h fixation, the cells were washed with PBS and stained with 20 µg/ml propidium iodide (PI) in PBS containing 20 µg/ml RNase A and incubated for 30 min at 37°C in the dark. Next, CytoFLEX flow cytometer (Beckman Coulter, Brea, United States) was used to quantify the DNA content in each sample. A total of 10,000 events were acquired for each sample, and the cell-cycle distribution was analyzed using ModFit LT 4.1 software (Verity Software House, Topsham, United States).

### Cell Counting Kit-8 Assays for Cell Viability

For the cell viability assay, the cells were seeded in 96-well plates at a density of 2,000 cells per well in normal growth medium (as described above) and incubated in 5% CO_2_ at 37°C. The following day, preadipocytes were transfected with pSDS-*Dnmt3a3* or pSDS as described above. The proliferation of the cell culture was monitored for three days at different time points (day 0, day 1, day 2, and day 3) using the TransDetect CCK kit (TransGen, Beijing, China) following the manufacturer’s instructions. Every 24 h, 10-µl of CCK-8 solution was added to each well and incubated in 5% CO_2_ at 37°C for 1 h.

The absorbance was measured using a Synergy™ Neo2 Multi-Mode Reader (Bio-Tek, Winooski, United States) by OD set at a wavelength of 450 nm.

### EdU (5-ethynyl-2′-deoxyuridine) Analysis

Cellular proliferation was assessed using the Cell-Light™ EdU Apollo567 *In Vitro* Kit (RiboBio, Guangzhou, China) according to the manufacturer’s instructions. Briefly, 210,000 preadipocytes were seeded in 6-well plates in normal growth medium (as described above) in 5% CO_2_ atmosphere at 37°C. The cells were grown to 60–70% confluence and transfected with Lipofectamine 3000 (Invitrogen). After transfection for 24 h, the cells were then grown for 24 h in normal growth medium containing EdU at 10 µM concentration. For each EdU experiment, three random fields were imaged using a Leica DMi8 confocal microscope (Leica) at 40× magnification. The images were analyzed in ImageJ v1.51j8 software (National Institutes of Health, Bethesda, United States). The EdU-positive cell number was counted as a percentage of the total cell number in each field using the Hoechst 33342 stain.

### Oil-Red-O Staining and Quantification

To determine fat accumulation, the cells were seeded in 12-well plates (19,000 cells per well) in normal growth medium (as described above). After 24 h, the cells were transfected with pSDS-*Dnmt3a3* or pSDS using Lipofectamine 3000 (Invitrogen). Six hours after transfection, the cells were switched to adipogenic medium (as described above). After a further 48 h, the cells were stained using Oil-Red-O staining kit (Solarbio) following the manufacturer’s protocol. Images were captured using a Leica DM2000 LED microscope (Leica). For the quantification of Oil-Red-O staining, the stain was extracted in 0.4 ml 100% isopropanol and 0.2 ml was used to measure Oil-Red-O stain in a 96-well plate at OD_510nm_.

### Western Blot Analysis

Protein lysate was prepared from the cells in ice-cold radioimmunoprecipitation assay buffer (Beyotime) containing 1 mM phenylmethylsulfonyl fluoride protease inhibitor. Protein concentration was measured using the BCA protein assay kit (Thermo Scientific). Standard electrophoresis was performed. For analysis of each specific protein, the protein lysate (20 µg) was separated on 12% sodium dodecyl sulfate-polyacrylamide gels (Beyotime) and transferred electrophoretically to polyvinylidene fluoride membranes (Beyotime). After blocking (QuickBlock™ reagent, Beyotime) for 30 min at room temperature, the membranes were incubated overnight at 4°C with the appropriate primary antibodies against p21 (1:1,000 dilution, GTX112898, GeneTex, Irvine, United States), p27 (1:200 dilution, bs-0742R, Bioss, Beijing, China), GATA3 (1:400 dilution, sc-268, Santa Cruz, California, United States), C/EBPβ (1:100 dilution, bs-1396R, Bioss), PPAR*γ* (1:500 dilution, bs-0530R, Bioss), and GAPDH (1:10,000 dilution, MB001H, Bioworld, St. Louis Park, United States). After washing five times (6 min once) in PBS with Tween-20 (PBST), the membranes were incubated with the appropriate secondary antibodies against peroxidase-conjugated goat anti-mouse IgG (1:10,000 dilution, BS12478, Bioworld) or peroxidase-conjugated goat anti-rabbit IgG (1:10,000 dilution, BS13278, Bioworld) for 1 h at room temperature with gentle agitation on a rocking plate. Signals were developed using a BeyoECL Star kit (Beyotime). Imaging of the bands was carried out using Odyssey Fc Imaging System (LI-COR, Nebraska, United States). Band intensity was quantified using ImageJ v1.51j8 software (National Institutes of Health).

### Statistical Analysis

Data analyses were performed using the SAS system for Windows V8 (SAS Institute, Cary, NC, United States) and GraphPad Prism 8.0.1 (GraphPad Software, San Diego, United States). The data are presented as the mean ± standard error (s.e.m.) from three independent experiments. Statistical comparisons were performed using One-way ANOVA followed by the Tukey test. In some experiments, Student’s *t*-test was performed to evaluate the significance of group differences. Probability (*P*) values of <0.05 were considered statistically significant.

## Results

### Identification of a Novel Spliced Variant of *Dnmt3a* in Chicken

**Figure 1:**
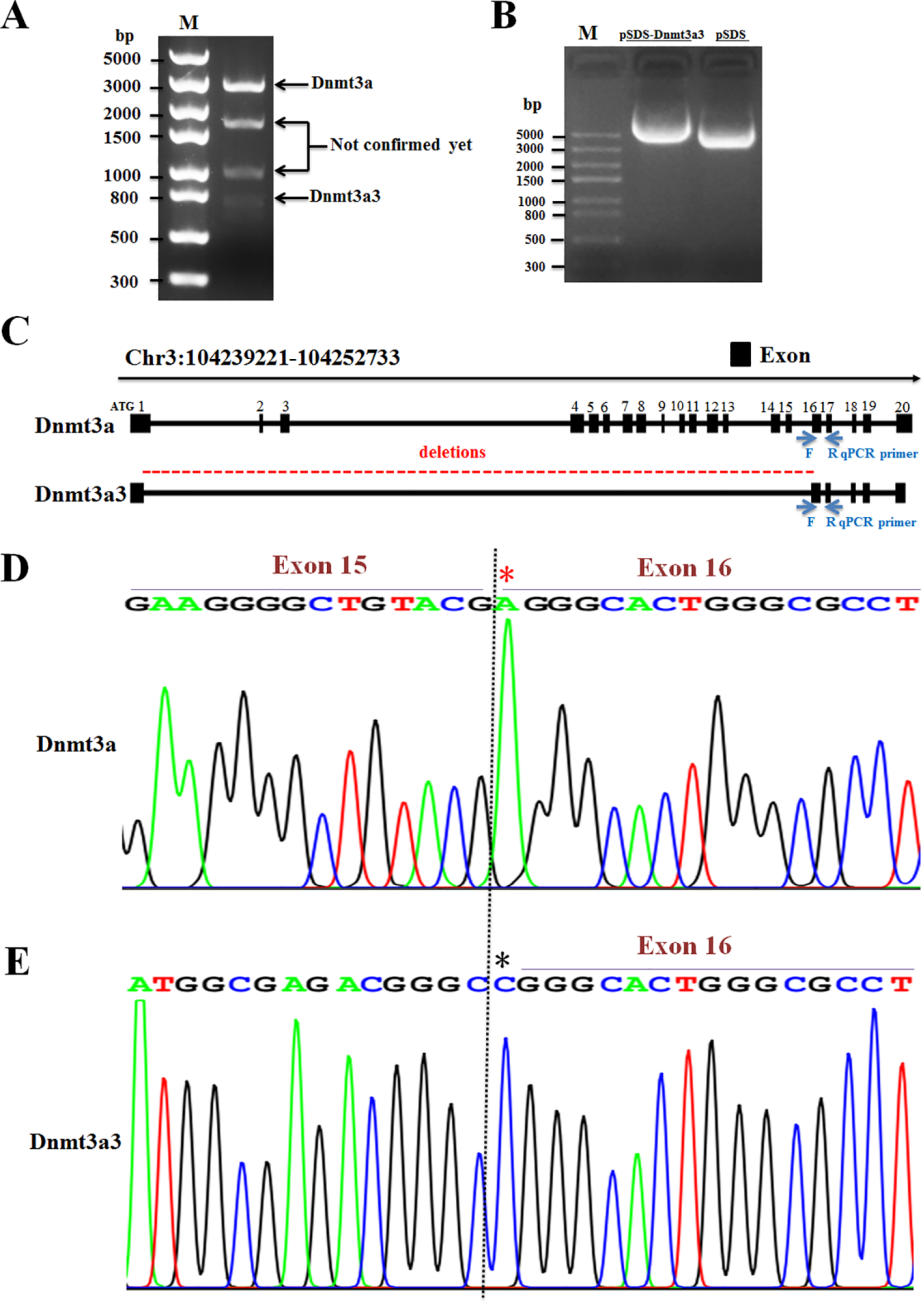
Identification of a novel spliced variant of chicken *Dnmt3a* gene. **(A)** The total RNA isolated from abdominal fat tissue of Huaixiang chicks was PCR amplified using the primer pair for *Dnmt3a* spliced variant detection (**Table 2**). The 1.5% agarose gel electrophoresis image of the amplified cDNA at around 3,000-bp (band No.1; *Dnmt3a*), 1,750-bp (band No.2), 1,000-bp (band No.3), and 800-bp (band No.4; *Dnmt3a3*) fragments is shown. The amplified product was subjected to sequencing analysis that identified a novel variant, named *Dnmt3a3*, which contains 809-bp (band No.4) and has been deposited in DDBJ: LC469746.1. M, DNA Marker; *Trans*5K DNA Ladder (TransGen). **(B)** As shown by the 1.5% agarose gel electrophoresis image, *Dnmt3a3* cDNA was successfully inserted into pSDS-25202 expression vector for constructing the *Dnmt3a3* overexpression plasmid. This constructed plasmid was confirmed by sequencing and named pSDS-*Dnmt3a3*. The pSDS-25202 empty vector was used as a control and named pSDS. M: *Trans*5K DNA Ladder (TransGen). **(C)** Structure of two spliced variants of chicken *Dnmt3a.* The obtained cDNA sequences were analyzed by BLAST. Blue arrows indicate the forward (F) and reverse (R) qRT-PCR primer locations for *Dnmt3a* and *Dnmt3a3*. The location in exon 16 contained a different sequence and, therefore, the primers are specific. A red, dashed line indicates exon sequence deletions. **(D)** Sanger sequencing of cDNAs showed that nucleotide A (red asterisk) in *Dnmt3a* exon 16 was replaced with nucleotide C (black asterisk) in *Dnmt3a3* exon 16 **(E)**, and exon 15 was deleted in *Dnmt3a3*.

**Figure 2:**
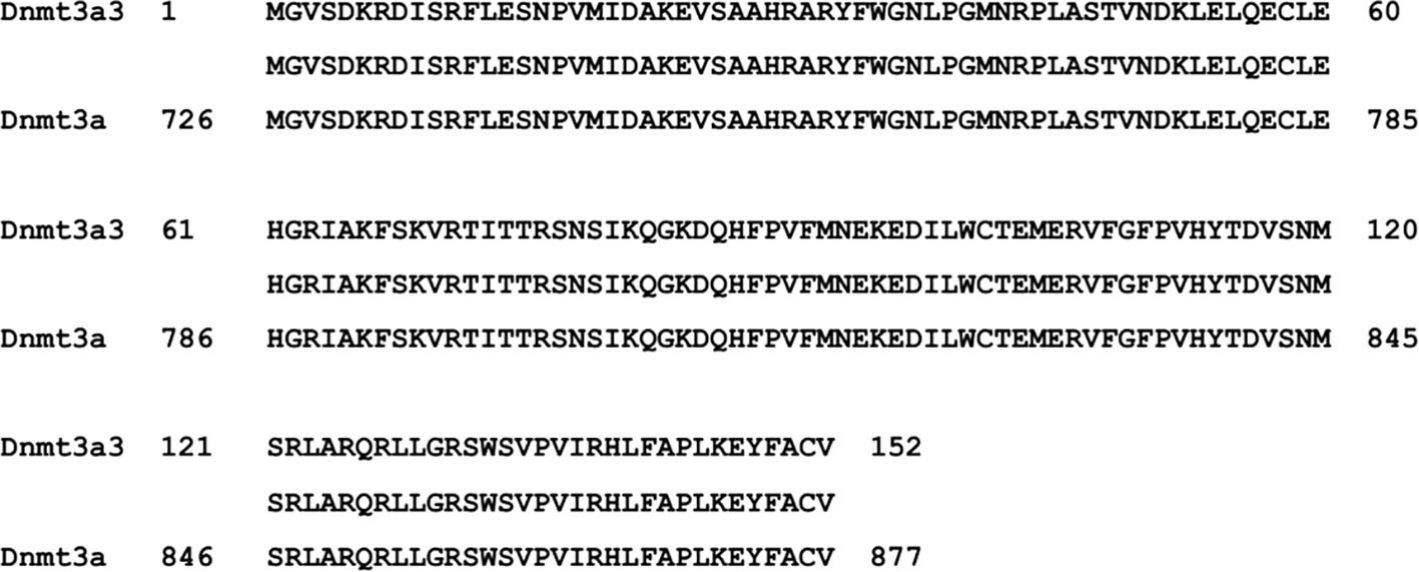
The possible ORF-alignment between Dnmt3a3 and Dnmt3a amino acids (aa). The initiator methionine (M) of the Dnmt3a3 protein started at 726 aa site of the Dnmt3a protein.

By cloning the full-length cDNA of the chicken *Dnmt3a* gene, one novel spliced variant (named *Dnmt3a3*) was successfully identified (**Figure 1A**; band no.4, **C–E)** and deposited into the DDBJ database with the accession number LC469746.1. According to the BLAST search results, the *Dnmt3a* variant (**Figure 1A**; band no.1, **C–E**) was similar to the one previously deposited into the NCBI (accession no. NM_001024832.1) and to our previously deposited accession number LC381635.1 (DDBJ database). Further analysis using the BLAST search (galGal4) revealed that the chicken *Dnmt3a* gene spanned over 13.5 kb at chromosome 3 (reverse strand) and consisted of 20 exons (**Figure 1C**). As shown in **Figures 1C–E**, exons from 2 to 15 of *Dnmt3a* were completely deleted from the *Dnmt3a3* transcript. About 120 bp were deleted in exon 1 of *Dnmt3a3*. The nucleotide A (red asterisk) in exon 16 of *Dnmt3a* was replaced with nucleotide C (black asterisk) in exon 16 of *Dnmt3a3*. The identified *Dnmt3a3* spliced variant was 809 bp in length (**Figure 1A**; band no.4) containing an open reading frame (ORF) of 456 bp and encodes a protein made up of 152 amino acids (**Figure 2**). The possible Dnmt3a3 protein was 725 aa (2,175 bp) shorter than Dnmt3a due to a 725 aa deletion.

### Expression of Chicken *Dnmt3a* Spliced Variants in Adipose Tissue and During Primary Preadipocyte Differentiation

**Figure 3:**
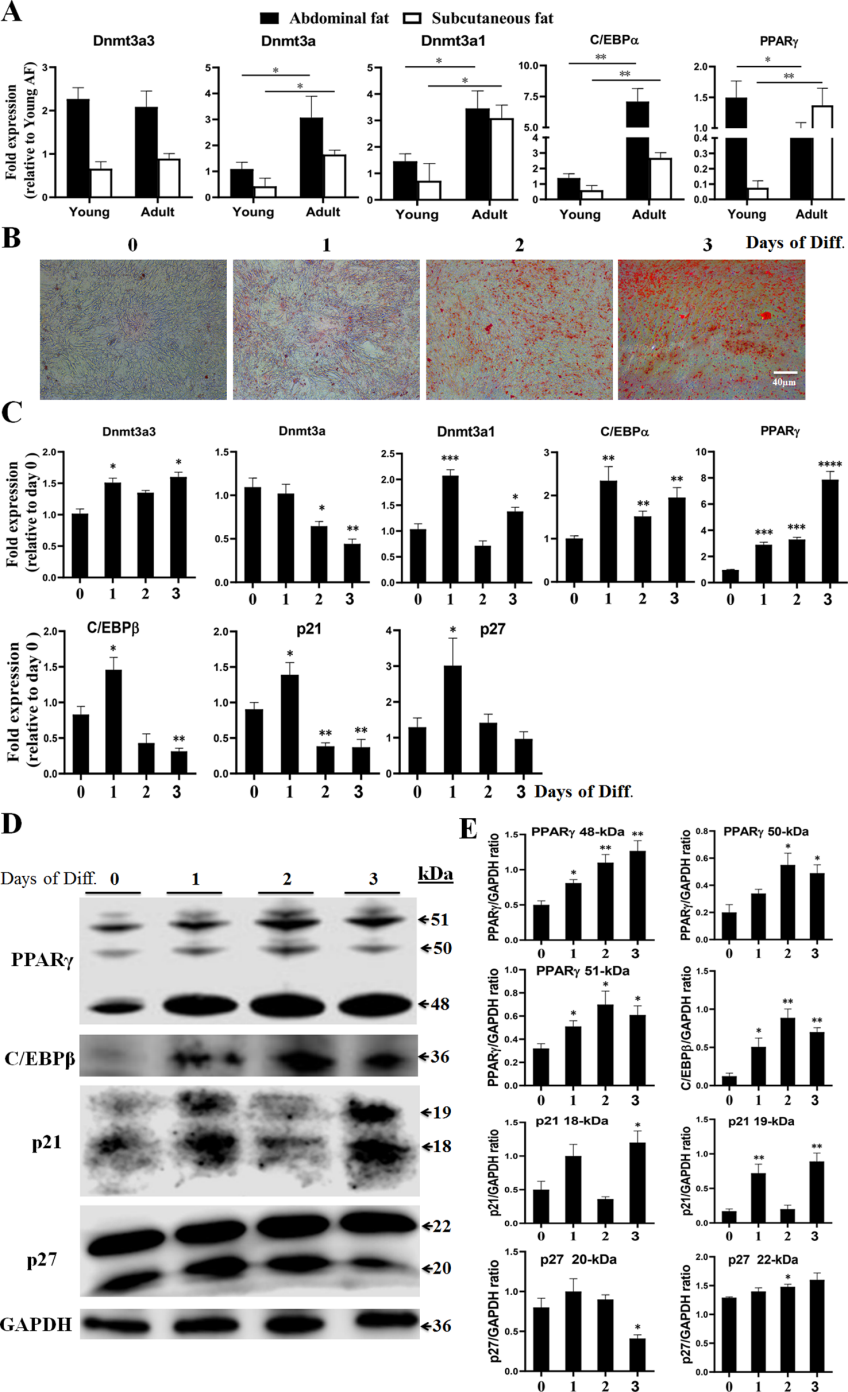
Expression of *Dnmt3a* spliced variants in adipose tissues and during the time-course analysis of preadipocyte differentiation. **(A)** Relative mRNA abundance of *Dnmt3a3*, *Dnmt3a*, *Dnmt3a1*, *C/EBPα*, and *PPARγ* in young and adult chicken adipose tissues. Values are expressed as mean ± s.e.m. (*P < 0.05 and **P < 0.01). AF, abdominal fat. **(B)** Oil-Red-O staining of chicken primary preadipocyte cells differentiated for three days. The Oil-Red-O stain is used to demonstrate adipocytes and neutral triglycerides. Images were taken at the indicated time points. Diff., differentiation. Scale bar: 40 µm. **(C)** The mRNA levels of *Dnmt3a* variant transcripts (*Dnmt3a3*, *Dnmt3a*, *and Dnmt3a1*), preadipocyte differentiation markers (*C/EBPα*, *PPARγ*, and *C/EBPβ*), and cell cycle control genes (*p21* and *p27*) were analyzed using qRT-PCR. Total RNA was extracted from the cells during differentiation at the indicated time points. Results are shown as mean ± s.e.m. (*P < 0.05, **P < 0.01, ***P < 0.001, and **** *P* < 0.0001). **(D)** Protein expression of PPAR*γ*, C/EBP*β*, p21, and p27 during the time-course analysis of preadipocyte differentiation. The protein expression was detected using Western blotting. The cells were harvested during differentiation at the indicated time points. The 48- and 51-kDa bands representing PPAR*γ*1 and PPAR*γ*2, respectively, and the 18-kDa band of p21 and 22-kDa band of p27, were illustrated. The other bands may be formed from alternative splicing. **(E)** Band intensity analysis of Western blot to calculate the level of each protein during differentiation was carried using ImageJ v1.51j8 software (National Institutes of Health). Values are expressed as mean ± s.e.m. (*P < 0.05 and **P < 0.01). GAPDH was used as the loading control.

To determine whether the *Dnmt3a3* transcript was expressed differentially in adipose tissues with increasing age, we analyzed *Dnmt3a3* mRNA expression levels in young and adult chicken adipose tissues using qRT-PCR. We isolated adipose tissues (abdominal fat and subcutaneous fat) from 25-day-old (hereafter termed young adipose tissues) and 350-day-old (hereafter termed adult adipose tissues) yellow-feathered chickens. Two transcription factors, C/EBPα and PPARγ, were used as specific markers for adipose tissue development (Spiegelman and Flier, 1996; Morrison and Farmer, 1999; Abdalla et al., 2018a; Ghaben and Scherer, 2019). The mRNA expression levels of chicken *Dnmt3a* and *Dnmt3a1* (Abdalla et al., 2018b) were also detected. As presented in **Figure 3A**, the mRNA expression level of *Dnmt3a3* showed no significant change between young adipose tissues and adult adipose tissues. However, the mRNA levels of *Dnmt3a* and *Dnmt3a1* were significantly down regulated in young adipose tissues. In addition, young adipose tissues showed decreased level of *C/EBPα* mRNA abundance relative to adult adipose tissues. Adult abdominal fat showed decreased level of *PPARγ* mRNA abundance relative to young abdominal fat, whereas adult subcutaneous fat showed the opposite results (**Figure 3A**).

In order to characterize the roles of *Dnmt3a3* on preadipocyte differentiation, the expression of *Dnmt3a3* during a time-course analysis of differentiation in primary preadipocytes was determined and compared with the expression of *Dnmt3a1* and *Dnmt3a*. Chicken primary preadipocyte was cultured in normal growth medium (as described under “MATERIALS AND METHODS”) until around 90% confluency. The growth medium was changed to differentiation medium (as described under “MATERIALS AND METHODS”) and the cells were allowed to differentiate for five days at different time points (day 0, day 1, day 2, day 3, day 4, and day 5). However, we observed that by day 4 of preadipocyte differentiation, 15% of the cells had died (data not shown). Therefore, we only used the data from day 0 to day 3 (day 0, day 1, day 2, and day 3) of differentiation. Oil-Red-O staining during the time-course analysis confirmed the increased rate and extent of differentiation (**Figure 3B**). The mRNA levels of *Dnmt3a3*, *Dnmt3a,* and *Dnmt3a1* at each day of differentiation was monitored by qRT-PCR. In order to monitor the differentiation process, the mRNA levels of specific adipose markers, such as *C/EBPα, PPARγ*, and *C/EBPβ,* and cell cycle control genes, such as *p21* and *p27,* were also measured using qRT-PCR. In addition, the protein levels of PPARγ, C/EBPβ, p21, and p27 were also monitored using Western blotting. qRT-PCR analysis (**Figure 3C**) indicates that the *Dnmt3a3* mRNA levels increased at the onset of differentiation and remained elevated throughout the three days of differentiation. The *Dnmt3a* mRNA expression level was significantly decreased in differentiated preadipocytes, whereas the *Dnmt3a1* mRNA level had peaked by day 1, then started to decrease by day 2, and increased again by day 3. During differentiation, PPARγ mRNA and protein levels were induced by day 1 and remained elevated throughout the course of differentiation (**Figures 3C–E**). *C/EBPα* is an early preadipocyte differentiation marker with a peak of expression that was detected by day 1 of differentiation. Meanwhile, *C/EBP*β is an early differentiation marker with a peak of expression that was detected by day 1 of differentiation followed by a sharp decline by days 2 and 3 of differentiation. The C/EBPβ protein level increased by day 1 and peaked by day 2 (**Figures 3C–E**). We also found that during early adipogenesis, the mRNA level of *p21* (a cell cycle regulator) peaked by day 1 of differentiation and decreased thereafter throughout the course of differentiation, whereas the protein level of p21 was elevated by day 1 and decreased by day 2, and again peaked by day 3. Similarly, the mRNA level of *p27*, another cell cycle regulator, peaked by day 1 of differentiation and decreased thereafter throughout the course of differentiation, whereas its protein level (22-kDa) gradually elevated throughout the course of differentiation (**Figures 3C–E**). Taken together, these results indicate that the mRNA level of *Dnmt3a3* was increased at the onset of preadipocyte differentiation and remained elevated throughout the time-course of differentiation. Thus, we focused on the biological effects of the *Dnmt3a3* variant on chicken primary preadipocyte proliferation and differentiation.

### *Dnmt3a3* Overexpression Inhibits Proliferation of Preadipocytes

**Figure 4:**
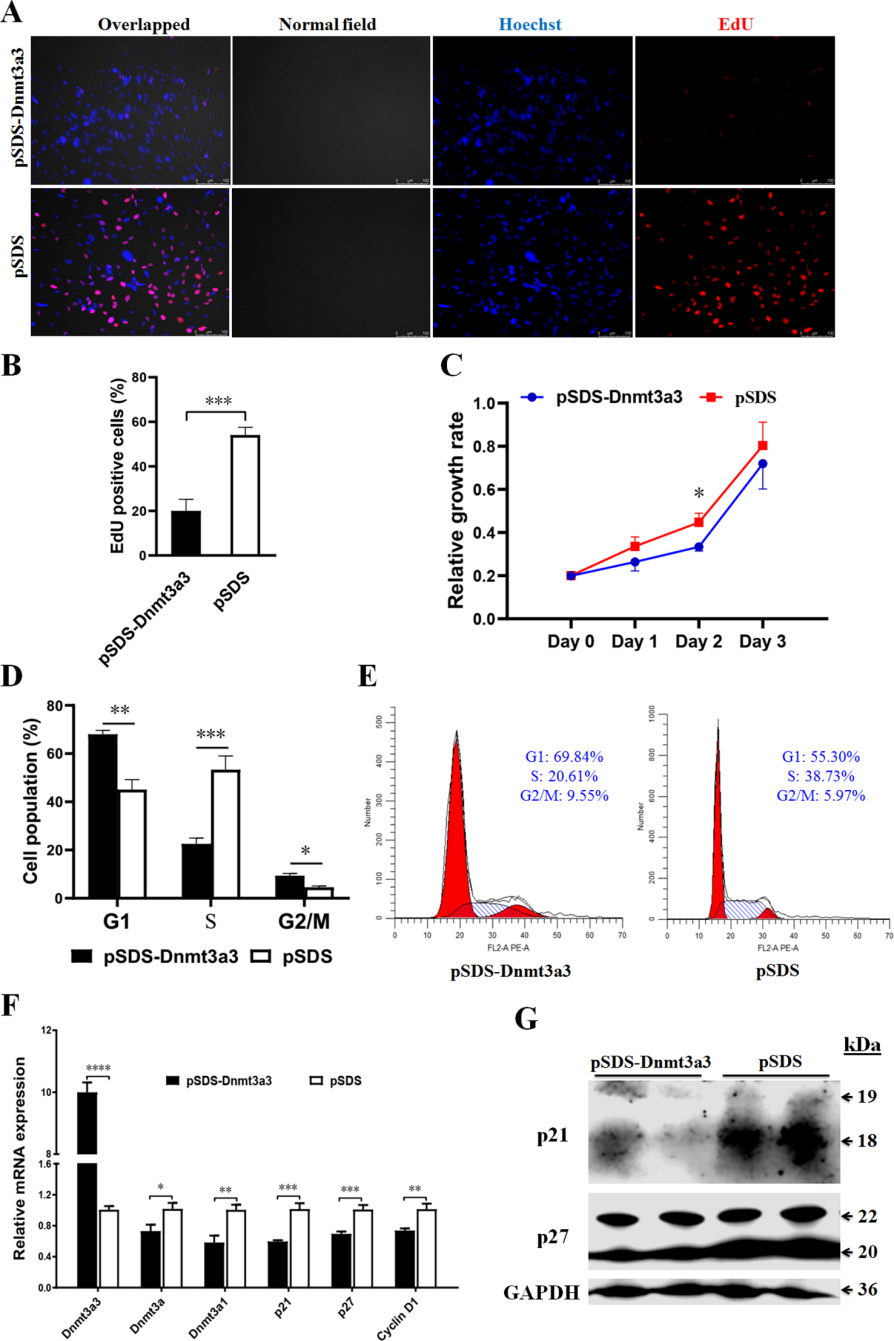
*Dnmt3a3* overexpression inhibits preadipocyte proliferation. **(A)** Representative images of EdU-stained cells. The cells were stained for EdU after transfection with pSDS-*Dnmt3a3* (*Dnmt3a3* overexpression) or pSDS (negative control). The EdU-positive nuclei are indicated in red. All the nuclei are indicated in blue (Hoechst). Scale bar: 100 µm. **(B)** Statistical analysis of EdU positive cells (%). Data are expressed as mean ± s.e.m. (***P < 0.001). **(C)** Cell viability was measured following the transfection of *Dnmt3a3* overexpression or negative control into primary preadipocytes. Values are expressed as mean ± s.e.m. (*P < 0.05). **(D)** Cell population (%) of preadipocytes after *Dnmt3a3* overexpression or negative control. The cell population was assessed using flow cytometric analysis. Data are expressed as mean ± s.e.m. (*P < 0.05, **P < 0.01, and ***P < 0.001). **(E)** Cell cycle analysis of preadipocytes after *Dnmt3a3* overexpression or negative control. A representative image of cell cycle distribution (G1, S, and G2/M) is indicated. **(F)** The mRNA levels of *Dnmt3a* transcripts (*Dnmt3a3*, *Dnmt3a*, and *Dnmt3a1*), several cell cycle markers (*p21*, *p27*, and *Cyclin D1*), and preadipocyte–adipocyte transition markers (*GATA2* and *GATA3*) after *Dnmt3a3* overexpression or negative control in proliferating preadipocytes. Results are shown as mean ± s.e.m. (*P < 0.05, **P < 0.01, ***P < 0.001, and ****P < 0.0001). **(G)** Protein expression of genes (p21 and p27) involved in cell cycle regulation after *Dnmt3a3* overexpression or negative control. GAPDH was used as the loading control.

**Figure 5:**
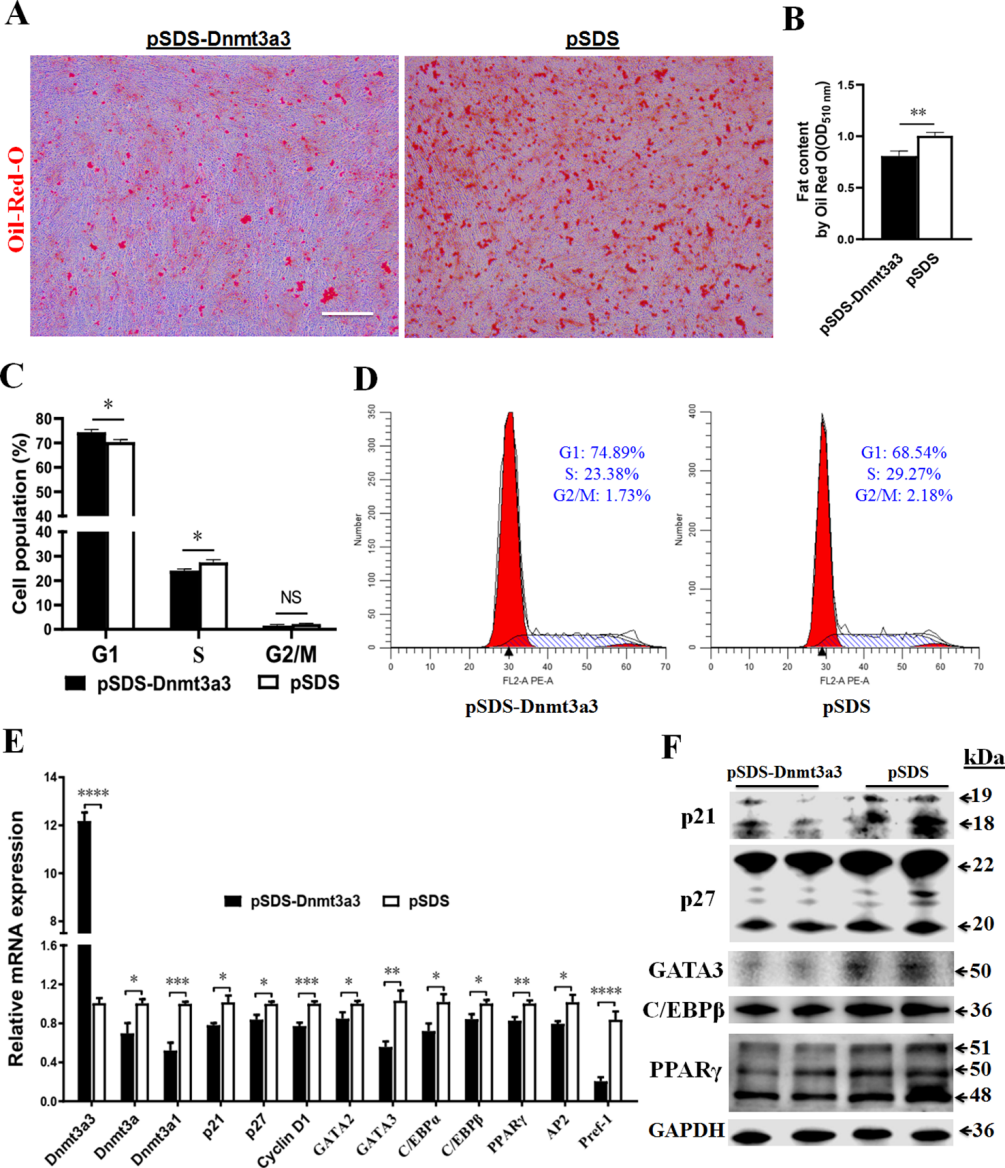
*Dnmt3a3* overexpression decreases fat accumulation in differentiating preadipocytes. Cultured preadipocytes were differentiated as described under “MATERIALS AND METHODS” **(A)** Oil-red-O stain was visualized following the transfection of *Dnmt3a3* overexpression (left panel) or negative control (right panel) into the cells. Images were captured at 10× magnification. Scale bar: 40 µm. **(B)** Quantification of Oil-Red-O staining after *Dnmt3a3* overexpression or negative control. The absorbance was measured at OD_510nm_ using a SynergyTM Neo2 Multi-Mode Reader (Bio-Tek). Values are expressed as mean ± s.e.m. (**P < 0.01). **(C)** Cell population (%) was determined following the transfection of *Dnmt3a3* overexpression or negative control. Values are expressed as mean ± s.e.m. (*P < 0.05). **(D)** Cell cycle analysis after *Dnmt3a3* overexpression or negative control. A representative image of cell cycle distribution (G1, S, and G2/M) is indicated. **(E)** The mRNA levels of chicken *Dnmt3a* transcripts (*Dnmt3a3*, *Dnmt3a*, and *Dnmt3a1*), several cell cycle control genes (*p21*, *p27*, and *Cyclin D1*), preadipocyte differentiation markers (*GATA2*, *GATA3*, *C/EBPα*, *C/EBPβ, PPARγ*, *AP2*, and *Pref-1*) were detected using qRT-PCR after *Dnmt3a3* overexpression or negative control. Values are expressed as mean ± s.e.m. (*P < 0.05, **P < 0.01, ***P < 0.001, and ****P < 0.0001). **(F)** Protein expression of cell cycle control markers (p21 and p27) and early preadipocyte differentiation markers (GATA3, C/EBP*β*, and PPAR*γ*) after *Dnmt3a3* overexpression or negative control in differentiating preadipocytes. GAPDH was used as the loading control.

In order to elucidate the biological roles of *Dnmt3a3* in preadipocyte proliferation, we conducted an overexpression experiment. A *Dnmt3a3* overexpression plasmid (pSDS-Dnmt3a3) was constructed (**Figure 1B**) and confirmed through sequencing. The *Dnmt3a3* mRNA expression levels in the cells were then modified through plasmid transfection for 48 hours with about tenfold overexpression, as quantified *via* qPCR-PCR (**Figures 4F, 5E**). After transfection for 48 hours, *Dnmt3a3* overexpression significantly suppressed preadipocyte proliferation, as judged by EdU assays (**Figures 4A, B**). We also monitored cell viability using CCK-8 assays and found that *Dnmt3a3* overexpression decreases the cell viability number (**Figure 4C**).

Given that *Dnmt3a3* inhibited preadipocyte proliferation, we sought to explore the effect of *Dnmt3a3* on cell cycle progression of preadipocytes. As expected, preadipocyte numbers in the G1 and G2/M phase were significantly elevated in *Dnmt3a3*-overexpressing preadipocytes compared with control preadipocytes, but were decreased in the S phase (**Figures 4D, E**). These data suggest that *Dnmt3a3* might inhibit preadipocyte proliferation by repressing the cell cycle transition at the G1/S phase and upregulating the G2/M phase.

In order to further understand the biological effects of *Dnmt3a3* on cell cycle progression, the mRNA level of several cell cycle-control genes, such as *p21*, *p27*, and *Cyclin D1*, were detected using qRT-PCR after *Dnmt3a3* overexpression in primary preadipocytes. We also detected the protein levels of p21 and p27 using Western blotting after *Dnmt3a3* overexpression. The results showed that overexpression of *Dnmt3a3* could inhibit the upregulation of cell cycle-control genes (**Figures 4F, G**). Taken together, these data points demonstrate that *Dnmt3a3* had a negative regulatory effect on chicken primary preadipocyte proliferation and cell cycle progression.

### *Dnmt3a3* Overexpression Represses Early Preadipocyte Differentiation

In order to elucidate the role of *Dnmt3a3* on early preadipocyte differentiation, we performed a *Dnmt3a3* overexpression experiment to assess the effect of *Dnmt3a3* on the cell cycle and cell differentiation processes. Compared to cells transfected with the control vector, stable primary preadipocytes overexpressing *Dnmt3a3* accumulated much less fat as determined by Oil-red-O staining after two days of differentiation (**Figure 5A**) or measured OD_510nm_ using the isopropanol method (**Figure 5B**).

Next, we asked whether *Dnmt3a3* overexpression affects the cell cycle program of differentiating preadipocytes. Using flow cytometric analysis, it was determined that *Dnmt3a3* overexpression resulted in an increase in the number of G1 phase cells but a clear decrease in the number of S phase cells was observed (**Figures 5C, D**). To gain further insight into the effects of *Dnmt3a3* overexpression on gene expression, the mRNA levels of cell cycle control genes, such as *p21*, *p27*, and *Cyclin D1*, were detected using qRT-PCR after overexpression of *Dnmt3a3.* Furthermore, the protein levels of p21 and p27 were detected using Western blotting. These results demonstrated that *Dnmt3a3* overexpression could repress the upregulation of cell cycle markers (**Figures 5E, F**). These data suggest that *Dnmt3a3* might inhibit differentiating preadipocyte cells by repressing the cell cycle transition at the G1/S phase.

To verify the role of *Dnmt3a3* on the chicken preadipocyte differentiation, the mRNA levels of early preadipocyte differentiation markers, such as *GATA2*, *GATA3*, *C/EBPα*, *C/EBPβ*, *AP2*, and *PPARγ* (Tong et al., 2000; Ghaben and Scherer, 2019), and *Pref-1*, whose expression is downregulated during adipocyte differentiation (Sul et al., 2000), were detected using qRT-PCR after overexpression of *Dnmt3a3.* Furthermore, the protein levels of GATA3, C/EBPβ, and PPARγ were also detected using Western blot analysis. The results showed that overexpression of *Dnmt3a3* can inhibit the upregulation of early differentiation markers (**Figures 5E, F**). Surprisingly, *Dnmt3a1* and *Pref-1* mRNA levels were also inhibited by *Dnmt3a3* overexpression. Interestingly, the mRNA levels of *Dnmt3a* decreased after overexpression of *Dnmt3a3* in proliferating and differentiating preadipocytes (**Figures 4F, 5E**). Collectively, these data indicate that *Dnmt3a3* negatively regulates preadipocyte cell growth and fat accumulation.

## Discussion

This study successfully identified one novel spliced variant of chicken *Dnmt3a* gene, termed *Dnmt3a3*. A BLAST search revealed that the alternative splicing event distinguishing *Dnmt3a3* from *Dnmt3a* occurred between exons 1 and 16. Exons 2 to 15 were completely deleted from the *Dnmt3a3* transcript. Due to splicing, both human and mouse *Dnmt3a* produce two different isoforms (Chen et al., 2002; Weisenberger et al., 2002). In this study (**Figure 1A**), we were unable to detect our previous chicken *Dnmt3a1* variant (Abdalla et al., 2018b) in pooled cDNA (four samples) from 25-day-old Huaixiang chicks. This suggests that the *Dnmt3a1* variant is not expressed in Huaixiang chicks at this developmental stage or that it may be expressed differently in different chicken breeds. Alternative splicing is a regulated process by which the exon/exons of pre-mRNA from a single gene can be spliced in different arrangements to generate structurally and functionally distinct mRNA and protein variants (Blencowe, 2006). In addition, exon skipping is the most common mode of alternative splicing in vertebrates and invertebrates (Kim et al., 2007). Compared with many organisms, chicken has a high rate of alternative splicing (Kim et al., 2007).

The high mRNA expression levels of *Dnmt3a3* in the abdominal fat tissue of young and adult chickens and during the time-course analysis of preadipocyte differentiation suggest that the enzyme functions in this system. Our results showed that *Dnmt3a3* and *Dnmt3a* expressed differently in young and adult adipose tissues and during the time-course analysis of preadipocyte differentiation. *Dnmt3a3* mRNA is abundantly expressed in young and adult abdominal fat tissues, while *Dnmt3a* mRNA abundance was markedly reduced in young adipose tissue. Thus, we propose that *Dnmt3a3* may regulate adipose tissue homeostasis in young and adult chickens. Our recent report demonstrated that *Dnmt3a1* was highly expressed in chicken adipose tissues and cells compared to *Dnmt3a* (Abdalla et al., 2018b). A previous study in mice demonstrated that *Dnmt3a* expression was increased in obese adipose tissues and cells compared to that of non-obese adipose tissues and cells (Kamei et al., 2010).

Our results confirm those of previous studies, which used oleate alone to induce preadipocyte differentiation in chickens (Liu et al., 2009; Shang et al., 2014). Chicken primary preadipocytes are capable of differentiating to adipocytes, as exhibited by the increase in mRNA and/or protein expression of early adipocyte differentiation markers, such as C/EBP*α*, C/EBP*β* and PPAR*γ* (Ntambi and Kim, 2000; Ghaben and Scherer, 2019), and the accumulation of lipid droplets by the cells (**Figures 3B–E**). It is well-known that the expression of C/EBP*α*, C/EBP*β*, and PPAR*γ* transcription factors induced during mouse 3T3-L1 adipogenesis (Morrison and Farmer, 1999; Tang et al., 2004). Also, the expression of p21 and p27 increased during 3T3-L1 early preadiocyte differentiation (Morrison and Farmer, 1999). In this study, during chicken primary preadiocyte differentiation, the expression of *Dnmt3a3, Dnmt3a1, C/EBPα*, *C/EBPβ*, *PPARγ, p21*, and *p27* mRNA levels increased at the onset of preadipocyte differentiation, while the *Dnmt3a* mRNA expression level decreased. This suggests that *Dnmt3a3* may also regulate adipogenesis in a manner similar to the *Dnmt3a1* transcript (Abdalla et al., 2018b). These results are in line with evidence indicating that the expression of murine *Dnmt3a* was downregulated during embryonic stem cell differentiation (Okano et al., 1998). Also, during the diﬀerentiation of mouse 3T3-L1 adipocytes, marked expression of adiponectin was observed, but *Dnmt3a* decreased at day 2 and there was almost no change from the initial observation by day 4 as differentiation progressed (Kamei et al., 2010).

In this study, we found that ectopic expression of *Dnmt3a3* inhibited preadipocyte proliferation. Preadipocytes are at a pivotal crossroads for adipocyte biogenesis. As they proliferate, they can either increase their population size or proceed to differentiate into mature adipocytes. Gene expression profiling of preadipocytes is closer to macrophages than mature adipocytes (reviewed by Tchkonia et al., 2010). Preadipocyte proliferation is associated with a number of cell cycle regulator genes (Morrison and Farmer, 1999; Zhu and Skoultchi, 2001). For example, CDks are serine/threonine kinases and their catalytic activities are modulated by interaction with Cdk inhibitors, such as p21 and p27 and Cyclins (Cyclin D1). The expression of p21, p27, and Cyclin D1 was altered between the proliferation and differentiation of 3T3-L1 fibroblasts from mice (Morrison and Farmer, 1999).

Preadipocyte differentiation requires many regulatory mechanisms that control cellular and molecular changes from satellite cell activation to terminal differentiation. Early preadipocyte differentiation requires the expression of a specific set of transcription factors, such as C/EBPβ, C/EBPα, and PPARγ, and the expression of several proteins, such as AP2 and GLUT4 (reviewed by Ghaben and Scherer, 2019). The *GLUT4*, an insulin-sensitive transporter, plays an important role in mice and rats (reviewed by Ghaben and Scherer, 2019), but it is not present within avian genomes. Moreover, *GATA2* and *GATA3* are found to control the preadipocyte–adipocyte transition (Tong et al., 2000). Our data demonstrated that *Dnmt3a3* overexpression repressed early preadipocyte differentiation, which is associated with decreased expression of GATA3, C/EBPβ, and PPARγ mRNA and protein levels, or *GATA2*, *C/EBPα*, and *AP2* mRNA levels. C/EBPβ expression is correlated with the expression of C/EBPα and PPARγ (Ntambi and Kim, 2000; Tang and Lane, 2012). The enhanced expression of *C/EBPβ* accelerates adipogenesis during early preadipocyte differentiation (Ntambi and Kim, 2000; Tang and Lane, 2012). Our previous study showed that overexpression of *Dnmt3a1* decreases PPARγ mRNA and protein levels, while inhibiting preadipocyte differentiation (Abdalla et al., 2018b). This direction of effect is consistent with the results of *Dnmt3a3*. Surprisingly, *Pref-1* (Sul et al., 2000) and *Dnmt3a1* (Abdalla et al., 2018b) are known to inhibit adipogenesis, but their mRNA expressions were downregulated by *Dnmt3a3* overexpression. This indicates that *Dnmt3a3* may be able to repress the expression of all regulators of adipogenesis. However, more work is needed to confirm this hypothesis. Mouse 3T3-L1 preadipocyte differentiation is strictly associated with cell cycle exit and was characterized by significant increases in p21 and p27 (Morrison and Farmer, 1999). RNA-mediated interference of p21 in 3T3-L1 fibroblasts reduced levels of C/EBP*α* and PPAR*γ*, and impaired adipocyte differentiation, resulting in smaller adipocytes (Inoue et al., 2008). Our results indicated that overexpression of *Dnmt3a3* during early differentiation of preadipocytes inhibited the upregulation of cell cycle control genes such as p21 and p27 mRNA and protein levels as well as *Cyclin D1* mRNA level, and this was accompanied by repression of the cell cycle transition at the G1/S phase. The expression of p21, regulated at the mRNA and protein levels, was directly associated with 3T3-L1 preadipocyte differentiation in mice (Morrison and Farmer, 1999). Furthermore, *Cyclin D1* overexpression acts in the G1 phase to enhance cell cycle progression in mammalian cells (Pestell et al., 1999). Cell cycle inhibition in the G1 phase may potentially trigger differentiation (Gonzales et al., 2015). Therefore, it will be interesting to determine if Dnmt3a3 can interact with the cell cycle control proteins p21, p27, and Cyclin D1 during the early stages of differentiation.

## Conclusions

This is the first report to identify a novel *Dnmt3a3* gene variant. Its mRNA level was increased at the onset of preadipocyte differentiation and remained elevated during the time course of differentiation. *Dnmt3a3* negatively regulates preadipocyte proliferation and differentiation. However, independent studies are necessary to confirm these data.

## Data Availability Statement

The datasets generated for this study can be found in the DDBJ/NCBI database (LC469746.1).

## Ethics Statement

The animal study was reviewed and approved by All experimental protocols were approved by the Institutional Animal Care and Use Committee of the South China Agriculture University.

## Author Contributions

BA conceived the project, designed the experiments, performed all experiments, and wrote the paper. ZL participated in the critical revising. QN participated in designing, revising, and coordination. All authors read and approved the final manuscript.

## Funding

This work was supported by grants from the Science and Technology Planning Project of Guangdong Province (2018B020203001), the National Natural Science Foundation of China (31950410539), the Ten Thousand Talents Program of China (W03020593), and the Talented Young Scientist Program of China (P18U44002).

## Conflict of Interest

The authors declare that the research was conducted in the absence of any commercial or financial relationships that could be construed as a potential conflict of interest.

## References

[B1] Abdalla B. A. Chen J. Nie Q. H. Zhang X. Q. (2018a). Genomic insights into the multiple factors controlling abdominal fat deposition in a chicken model. Front. Genet. 9 262. 10.3389/fgene.2018.00262 30073018 PMC6060281

[B2] Abdalla B. A. Li Z. Ouyang H. Jebessa E. Sun T. Yu J. A. (2018b). A Novel Dnmt3a1 transcript inhibits adipogenesis. Front. Physiol. 9, 1270. 10.3389/fphys.2018.01270 30333755 PMC6176318

[B3] Blencowe B. J. (2006). Alternative splicing: new insights from global analyses. Cell 126, 37–47. 10.1016/j.cell.2006.06.023 16839875

[B4] Brett D. Pospisil H. Valcarcel J. Reich J. Bork P. (2002). Alternative splicing and genome complexity. Nat. Genet. 30, 29–30. 10.1038/ng803 11743582

[B5] Chen T. Ueda Y. Xie S. Li E. (2002). A novel Dnmt3a isoform produced from an alternative promoter localizes to euchromatin and its expression correlates with active *de novo* methylation. J. Biol. Chem. 277, 38746–38754. 10.1074/jbc.M205312200 12138111

[B6] Ghaben A. L. Scherer P. E. (2019). Adipogenesis and metabolic health. Nat. Rev. Mol. Cell Biol. 20, 242–258. 10.1038/s41580-018-0093-z 30610207

[B7] Gonzales K. A. Liang H. Lim Y. S. Chan Y. S. Yeo J. C. Tan C. P. (2015). Deterministic restriction on pluripotent state dissolution by cell-cycle pathways. Cell 162, 564–579. 10.1016/j.cell.2015.07.001 26232226

[B8] Inoue N. Yahagi N. Yamamoto T. Ishikawa M. Watanabe K. Matsuzaka T. (2008). Cyclin-dependent kinase inhibitor, p21(WAF1/CIP1), is involved in adipocyte differentiation and hypertrophy, linking to obesity, and insulin resistance. J. Biol. Chem. 283, 21220–21229. 10.1074/jbc.M801824200 18445590 PMC3258954

[B9] Kamei Y. Suganami T. Ehara T. Kanai S. Hayashi K. Yamamoto Y. (2010). Increased expression of DNA methyltransferase 3a in obese adipose tissue: studies with transgenic mice. Obesity 18, 314–321. 10.1038/oby.2009.246 19680236

[B10] Kim E. Magen A. Ast G. (2007). Different levels of alternative splicing among eukaryotes. Nucleic Acids Res. 35, 125–131. 10.1093/nar/gkl924 17158149 PMC1802581

[B11] Liu S. Wang L. Wang N. Wang Y. X. Shi H. Li H. (2009). Oleate induces transdifferentiation of chicken fibroblasts into adipocyte-like cells. Comp. Biochem. Phys. A 154, 135–141. 10.1016/j.cbpa.2009.05.011 19470408

[B12] Morrison R. F. Farmer S. R. (1999). Role of PPARgamma in regulating a cascade expression of cyclin-dependent kinase inhibitors, p18(INK4c) and p21(Waf1/Cip1), during adipogenesis. J. Biol. Chem. 274, 17088–17097. 10.1074/jbc.274.24.17088 10358062

[B13] Ntambi J. M. Kim Y. C. (2000). Adipocyte differentiation and gene expression. J. Nutr. 130, 3122s–3126s. 10.1093/jn/130.12.3122S 11110885

[B14] Okano M. Xie S. Li E. (1998). Cloning and characterization of a family of novel mammalian DNA (cytosine-5) methyltransferases. Nat. Genet. 19, 219–220. 10.1038/890 9662389

[B15] Okano M. Bell D. W. Haber D. A. Li E. (1999). DNA methyltransferases Dnmt3a and Dnmt3b are essential for *de novo* methylation and mammalian development. Cell 99, 247–257. 10.1016/s0092-8674(00)81656-6 10555141

[B16] Pestell R. G. Albanese C. Reutens A. T. Segall J. E. Lee R. J. Arnold A. (1999). The cyclins and cyclin-dependent kinase inhibitors in hormonal regulation of proliferation and differentiation. Endocr. Rev. 20, 501–534. 10.1210/edrv.20.4.0373 10453356

[B17] Shang Z. Guo L. Wang N. Shi H. Wang Y. Li H. (2014). Oleate promotes differentiation of chicken primary preadipocytes *in vitro* . Biosci. Rep. 34 51–57. 10.1042/BSR20130120 PMC3917231 27919046

[B18] Siegel P. B. (2014). Evolution of the modern broiler and feed efficiency. Annu. Rev. Anim. Biosci. 2, 375–385. 10.1146/annurev-animal-022513-114132 25384148

[B19] Spiegelman B. M. Flier J. S. (1996). Adipogenesis and obesity: rounding out the big picture. Cell 87, 377–389. 10.1016/s0092-8674(00)81359-8 8898192

[B20] Sul H. S. Smas C. Mei B. Zhou L. (2000). Function of pref-1 as an inhibitor of adipocyte differentiation. Int. J. Obesity 24, S15–S19. 10.1038/sj.ijo.0801494 11126233

[B21] Tang Q. Q. Lane M. D. (2012). Adipogenesis: from stem cell to adipocyte. Annu. Rev. Biochem. 81, 715–736. 10.1146/annurev-biochem-052110-115718 22463691

[B22] Tang Q. Q. Zhang J. W. Lane M. D. (2004). Sequential gene promoter interactions by C/EBP beta, C/EBP alpha, and PPAR gamma during adipogenesis. Biochem. Bioph. Res. Co. 318, 213–218. 10.1016/j.bbrc.2004.04.017 15110775

[B23] Tchkonia T. Morbeck D. E. Von Zglinicki T. Van Deursen J. Lustgarten J. Scrable H. (2010). Fat tissue, aging, and cellular senescence. Aging Cell 9, 667–684. 10.1111/j.1474-9726.2010.00608.x 20701600 PMC2941545

[B24] Tong Q. Dalgin G. Xu H. Ting C. N. Leiden J. M. Hotamisligil G. S. (2000). Function of GATA transcription factors in preadipocyte-adipocyte transition. Science 290, 134–138. 10.1126/science.290.5489.134 11021798

[B25] Wang X. Yang M. Ren D. Terzaghi W. Deng X. W. He G. (2019). Cis-regulated alternative splicing divergence and its potential contribution to environmental responses in Arabidopsis. Plant J. 97, 555–570. 10.1111/tpj.14142 30375060

[B26] Weisenberger D. J. Velicescu M. Preciado-Lopez M. A. Gonzales F. A. Tsai Y. C. Liang G. N. (2002). Identification and characterization of alternatively spliced variants of DNA methyltransferase 3a in mammalian cells. Gene 298, 91–99. 10.1016/S0378-1119(02)00976-9 12406579

[B27] Zhu L. Skoultchi A. I. (2001). Coordinating cell proliferation and differentiation. Curr. Opin. Genet. Dev. 11, 91–97. 10.1016/S0959-437X(00)00162-3 11163157

